# Evidence-based, Skin-directed Treatments for Cutaneous Chronic Graft-versus-host Disease

**DOI:** 10.7759/cureus.6462

**Published:** 2019-12-25

**Authors:** Yoo Jung Kim, Gun Ho Lee, Bernice Y Kwong, Kathryn J Martires

**Affiliations:** 1 Department of Dermatology, Stanford University School of Medicine, Stanford, USA; 2 Department of Dermatology, Palo Alto Medical Foundation, San Jose, USA

**Keywords:** chronic, hematopoietic stem cell transplantation, hematopoietic stem cells, skin, therapy, graft versus host disease (gvhd), chronic graft versus host disease (cgvhd)

## Abstract

Chronic graft-versus host disease (cGVHD) occurs in 30% to 70% of patients undergoing allogeneic hematopoietic cell transplantation (HCT). Cutaneous cGVHD affects 75% of cGVHD patients, causing discomfort, limiting the range of movement, and increasing the risk of wound infections. Furthermore, systemic immunosuppression is often needed to treat cGVHD and long-term use can lead to adverse events. Optimal use of skin-directed therapies is integral to the management of cutaneous cGVHD and may decrease the amount of systemic immunosuppression required.

This study reviewed English-language articles published from 1990 to 2017 that evaluated the effect of skin-directed treatments for cutaneous cGVHD. A total of 201 papers were identified, 164 articles were screened, 46 were read, and 18 publications were utilized in the review. Skin-directed treatments for cGVHD included topical steroids, topical calcineurin inhibitors, psoralen with ultraviolet A (PUVA) irradiation, ultraviolet A1 (UVA1) irradiation, and ultraviolet B (UVB) irradiation. We report the number of complete remissions, partial remissions, and systemic immunosuppression reduction in each study, as available.

Twenty-two out of 30 (73.3%) patients experienced overall improvement with topical calcineurin inhibitors. At least 26 out of 76 patients (34.2%) receiving PUVA experienced complete remission, and 30 out of 76 patients (39.5%) experienced partial remission. In UVA1 studies, 44 out of 52 (84.6%) patients experienced overall improvement. In UVB studies, nine out of 14 patients (64.3%) experienced complete remission and four out of 14 patients (28.6%) experienced partial remission.

As more HCTs are performed, more individuals will develop cGVHD. Awareness and optimal use of skin-directed therapies for cutaneous cGVHD may help improve patient outcomes and quality of life.

## Introduction and background

Chronic graft-versus-host disease (cGVHD) occurs in 30% to 70% of post-hematopoietic cell transplantation (HCT) patients [1-2]. According to the Health Resources and Services Administration, in 2017, about 23,000 umbilical cord blood and bone marrow transplants were performed in the United States. Compared to non-transplanted, case-matched controls, 10-year survivors of allogeneic HCT report a poorer quality of life, including greater discomfort or dysfunction during sexual activity, memory problems, and higher rates of antidepressant and anxiolytic use [3]. These issues are likely associated with cGVHD symptoms. Chronic GVHD is a type IV hypersensitivity reaction, which occurs when donor effector T-cells from the graft recognize the cells of the recipient as foreign [4]. Control of cGVHD is integral for preventing morbidity and mortality in allogeneic HCT patients, and supportive measures, including skin-directed therapies, can improve skin symptoms and quality of life in post-allogeneic HCT patients.

The skin is the most commonly involved organ in cGVHD, with cutaneous cGVHD occurring in approximately 75% of cGVHD patients [1]. Cutaneous manifestations of cGVHD are associated with pruritus and pain, limited range of motion, and increased risk of wound infection [5]. Skin-directed therapies may improve control of skin disease and quality of life in patients without incurring the adverse effects of systemic immunosuppressive treatment.

**Figure 1:**
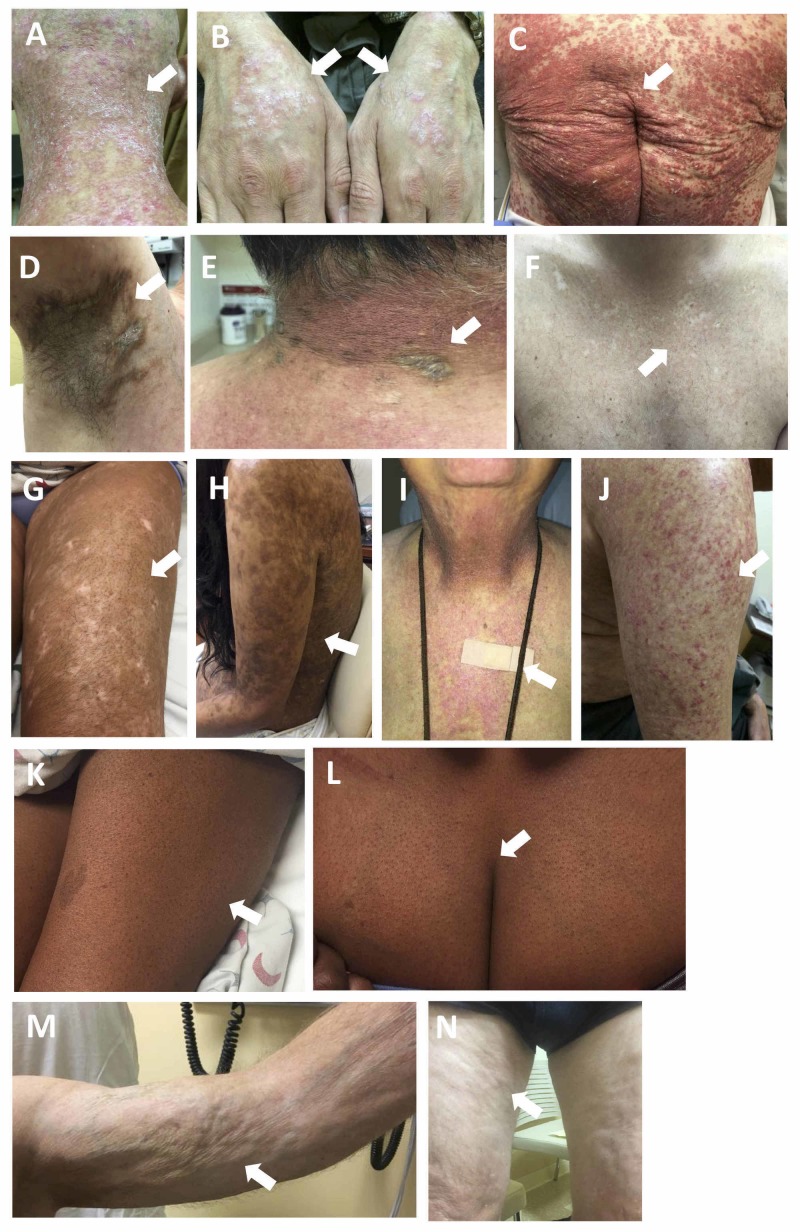
Examples of cutaneous chronic graft-versus-host disease morphologies A, B: lichen planus-like; C: papulosquamous-like; D: lichen sclerosus-like; E: morphea-like; F, G, H: dyspigmentation; I, J: poikilodermatous; K, L: keratosis pilaris-like; M, N: dermal and subcutaneous skin changes

Cutaneous cGVHD has historically been categorized as lichen planus-like or sclerotic types; now, skin manifestations of cGVHD are understood to have highly variable morphologies. The 2014 International National Institutes of Health Chronic GVHD Diagnosis and Staging Consensus Working Group suggested the following clinical signs for diagnosing cutaneous cGVHD: poikiloderma, lichen planus-like, sclerotic, morphea-like, and lichen sclerosus-like features [6]. Other findings include dyspigmented, eczematous, vitiligo-like, alopecia, and papulosquamous lesions (Figure 1). More rare morphologies of GVHD include a thick-appearing white tongue, inverse pityriasis rosea-like, eczema-like features, and follicular hyperkeratosis [7].

The first-line treatment for cutaneous cGVHD includes systemic corticosteroids. However, corticosteroids produce long-term responses in about half of cGVHD patients [8] and are unsuitable for long-term use due to serious adverse effects [9]. The use of topical or skin-directed treatments should supplement systemic steroid therapy. Low-grade manifestations of skin cGVHD may be amenable to local, skin-directed treatment [6]. Topical, non-systemic therapies can improve patients' quality of life and allow for immunosuppression tapering.

As advances in HCT methods improve mortality rates for post-transplant patients, the number of patients facing long-term effects of HCT, including cutaneous cGVHD, will grow [10]. Multiple systemic and topical therapies are available for cutaneous GVHD, and this study aims to provide an overview of available skin-directed treatment for cutaneous manifestations of cGVHD [11-12].

## Review

Methods

**Figure 2:**
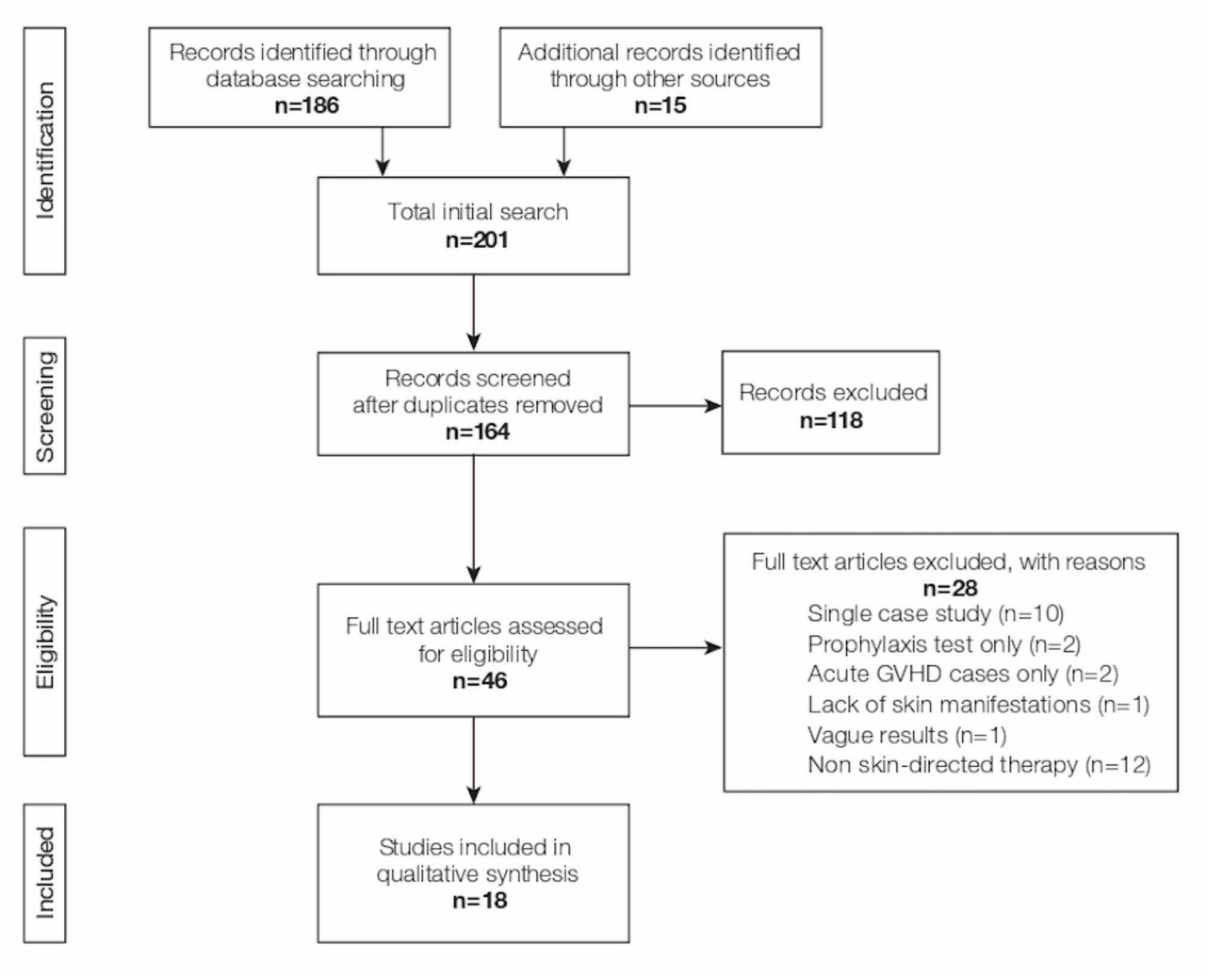
PRISMA Flow Diagram GVHD: graft-versus-host disease; PRISMA: Preferred Reporting Items for Systematic Reviews and Meta-analyses

We searched PubMed to find English-language articles from 1990 to 2017 evaluating the efficacy of skin-directed treatments in patients exhibiting cutaneous cGVHD (Figure 2). We included articles evaluating topical steroids, topical calcineurin inhibitors, psoralen in combination with UVA (PUVA), ultraviolet A1 (UVA1) irradiation, and ultraviolet B (UVB) irradiation. The search was conducted using the following terms: “chronic GVHD topical treatment” and “chronic GVHD skin (name of treatment).” The reference lists in the selected studies were reviewed to identify additional articles. No limits were applied in the initial search. We excluded articles that contained fewer than two patients, focused solely on cGVHD prophylaxis, studied only systemic therapies, or lacked skin-specific results. A review of the literature revealed no studies examining the use of topical or intralesional steroids alone in cGVHD patients. Therefore, we added a section on topical steroids describing our institution’s practices.

After reviewing the selected articles, we extracted the following data in a *post hoc* analysis: type of cutaneous cGVHD, treatment protocol, average cumulative irradiation in phototherapy studies, complete resolution (CR), partial resolution (PR), overall improvement (OI), concomitant immunosuppression, reduction in immunosuppression, and adverse effects. CR was as classified in the study or determined from the proportion of patients experiencing complete resolution of skin symptoms. PR was as classified in the study or determined from the proportion of patients experiencing less-than-complete/unspecified improvement in skin symptoms. OI was calculated as the number of patients presenting with any improvement in symptoms.

Some studies included patients who were not relevant to the scope of our review, including acute GVHD patients and cGVHD patients without skin disease; these studies were labeled with an asterisk (*) in the included tables [13-19]. Whenever feasible, only cGVHD patients with cutaneous symptoms were counted toward the number of patients in each study with CR, PR, OI, and reduction in immunosuppression. If a study did not provide enough information to exclude non-relevant patients from the calculations above, this was also noted in the tables.

Topical steroids 

Topical steroids are integral for managing cutaneous cGVHD, especially for the ichthyotic, papulosquamous, lichen planus-like, and lichen sclerosus-like forms. Even for focal morphea-like and other forms of sclerotic cGVHD, topical steroids can be beneficial. Topical steroids have many effects on the skin, including decreasing epidermal inflammatory cells, dendritic cell responses, synthesis of pro-inflammatory factors, and the production and cross-linking of collagen. Intralesional steroids may also be considered.

The choice of a topical steroid, vehicle, and the regimen employed can be highly variable and depends on many factors, such as the anatomic region, level of the skin (epidermis vs. dermis vs. subcutaneous) affected, and expected patient compliance. As a general rule, for thinner skin areas (including the face, neck, axillae, and groin), a low-potency topical steroid should be employed, such as hydrocortisone 2.5%, fluocinolone 0.01%, or triamcinolone 0.025%. The scalp is an exception to this rule, as higher-potency steroids may be employed if necessary; furthermore, steroid solutions or oils can be used to aid with the application in the scalp.

Ointments are typically more potent than creams. For epidermal forms of cGVHD, such as ichthyosiform, lichenoid, and papulosquamous, triamcinolone 0.1% ointment can be prescribed. For extensive involvement, a 1-pound (454 gram) jar can be provided, as dispensing adequate amounts of topical steroids is essential for proper treatment.

For lichen sclerosus and sclerotic forms of cGVHD, higher potency Class 1 (e.g., clobetasol propionate 0.05%) or Class 2 (e.g., fluocinonide 0.05%) topical steroids should be considered as first-line therapies, especially in cases where the lesions are active or progressing. Topical steroids are typically used twice daily. While ointments are more effective, many patients prefer creams due to the ease of use. In these cases, a cream can be used during the day for practical use with clothing and an ointment at night for better occlusion. For focal sclerotic disease, topical steroids can be applied under occlusion with plastic wrap for increased efficacy. In cases with extensive body surface area involvement, a technique called the “soak and smear" may be employed. This regimen involves a 20-minute soak in plain warm water, followed by the application of generous amounts of topical steroid ointment by a “smearing” technique. Sauna suits or pajamas are then worn overnight to maximize the absorption of topical steroids through occlusion [20].

The adverse effects associated with topical steroids include atrophy, blood vessel dilation, and steroid acne. Prolonged use of the aforementioned “soak and smear” regimen may result in systemic absorption of the topical agent. 

Topical tacrolimus

Systemic tacrolimus is used as systemic prophylaxis and treatment for cGVHD. Tacrolimus binds to FK506, leading to the inhibition of calcineurin. Calcineurin is then unable to activate the transcription factor nuclear factor of activated T-cells (NFAT), preventing the expression of interleukin-2 and other key cytokines of immune activation [21].

Topical tacrolimus ointment is widely used as a steroid-sparing agent for atopic dermatitis. By decreasing cytokine expression in the skin, topical tacrolimus may improve the appearance and symptoms associated with sclerotic and non-sclerotic skin cGVHD lesions.

In three studies, 30 patients with cutaneous cGVHD received topical tacrolimus ointments (Table 1) [22-24]. Twenty-two patients (73.3%) demonstrated some degree of improvement. Reduction in systemic immunosuppression was not described in any of the studies.

**Table 1:** Studies Describing Topical Tacrolimus Use for Cutaneous Chronic Graft-versus-host Disease (cGVHD) CR: complete remission; IV: intravenous; OI: overall improvement; PR: partial remission; UVB: ultraviolet-B

Author, Year	Type of cGVHD	Treatment Protocol	Study Size	CR	PR	OI	Concomitant Immunosuppression	Reduction in Immunosuppression	Adverse Effects
Choi and Nghiem [22]	Not specified	0.1% tacrolimus ointment 2 - 3x/day. Discontinued if no improvement or adverse effects	18	—	—	13	16/18 prednisone, 14/18 cyclosporine, 5/18 mycophenolate mofetil, 1/18 dexamethasone	—	Uncomfortable sensation (1/18)
Elad et al. [23]	Lichenoid, sclerodermatous	0.03 - 0.1% tacrolimus ointment 2 - 3x/day.	10	—	—	7	7/10 azathioprine, 6/10 cyclosporine, 3/10 methotrexate, 2/10 fludarabine, 2/10 thalidomide, 2/10 UVB	Systemics unchanged during treatment by design	Burning sensation (1/10)
Olson et al. [24]	Not specified	0.1% tacrolimus ointment 2x/day with occlusion	2	—	—	2	2/2 topical corticosteroid, 2/2 oral tacrolimus, 1/2 oral corticosteroid, 1/2 IV corticosteroid, 1/2 IV rituximab, 1/2 photopheresis	—	Irregular systemic absorption of tacrolimus (2/2)

Choi and Nghiem published a case series of 18 cGVHD patients treated with 0.1% topical tacrolimus ointment [22]. Thirteen patients experienced improvement in pruritus or erythema within “hours to days” of initiating treatment. However, all patients also required additional therapies, such as increased dosages of corticosteroids, PUVA, or extracorporeal phototherapy, leading the authors to conclude that topical tacrolimus should be used as an adjunct treatment.

Elad et al. published similar findings in a study showing limited skin improvement in seven out of 10 patients receiving 0.03% - 0.1% tacrolimus ointment two to three times daily [23]. The examiner reported skin improvements within a day of tacrolimus administration.

Olson et al. presented a case report of two patients with erythematous cutaneous cGVHD treated with tacrolimus 0.1% ointment with occlusive dressings twice a day [24]. Both patients improved overall while being concomitantly treated with oral tacrolimus, systemic corticosteroids, and topical corticosteroids. However, the authors noted significant systemic tacrolimus absorption leading to erratic tacrolimus troughs, ultimately resulting in the cessation of topical tacrolimus in both patients.

In comparison, in the Choi/Nghiem and Elad et al. studies, adverse effects were limited to local discomfort at the site of tacrolimus administration [22-23]. Other studies observing tacrolimus use on the treatment of atopic dermatitis noted that a burning sensation and skin flushing were common adverse events [25-26].

In contrast to corticosteroids, tacrolimus does not affect collagen synthesis and can be used where atrophy is of particular concern--the face, flexural surfaces, axillae, etc. [27]. In previous studies studying atopic dermatitis, the bioavailability of 0.3% tacrolimus ointment was less than 0.5% that of IV tacrolimus and less than 5% that of oral tacrolimus; however, the erratic systemic absorption, as shown in the Olson study, demonstrates that more research needs to be done regarding the safety of tacrolimus in the cGVHD patient population [24-25, 28].

One potential concern regarding calcineurin inhibitor treatment is the black box warning for topical tacrolimus regarding the risk of lymphoma. Systemic tacrolimus has been associated with an increased risk of malignancies, and questions were raised regarding the safety of topical tacrolimus [29]. To estimate the risk of topical tacrolimus use on the development of cancer and lymphoma, researchers conducted a cohort study of 19,948 children and 66,127 adults starting tacrolimus. Among children, there were five events of lymphoma out of 47,872 person-years, and among children taking corticosteroids, there were four events of lymphoma in 191,074 person-years. The incidence rate ratio of developing lymphoma for topical tacrolimus versus topical corticosteroids was 3.74 (95% confidence interval: 1.00 - 14.06) for children and 1.27 (95% confidence interval: 0.94 - 1.71) for adults [30]. The authors stated, "the low (incident rate ratio) implies that even if the increased risk is causal, it represents a small excess risk for individual patients." It is generally accepted among dermatologists that topical calcineurin inhibitors are a safe alternative to topical steroids.

Psoralen in combination with UVA (PUVA)

Psoralen in combination with UVA (UVA: 320 - 400 nm), also known as PUVA, is used to treat a variety of skin conditions, including psoriasis, lichen planus, and cutaneous T-cell lymphoma [12]. When activated by UVA, psoralen covalently binds and crosslinks deoxyribonucleic acid (DNA) base pairs, inhibiting cell proliferation and causing immunosuppression by mechanisms that are not fully understood.

Clinical administration of 8-methoxypsoralen (8-MOP) in PUVA treatment occurs in two ways. One involves the ingestion of 8-MOP at least an hour before irradiation, and the other involves topical application via a 20-minute bath immersion in a solution of 8-MOP before UVA treatment. Of note, protective eyewear needs to be worn for 24 hours after taking oral 8-MOP. Patients are treated two to four times per week, and the UVA irradiation dose can be increased based on patient tolerance and improvement of symptoms. In our review, the cumulative irradiation doses for PUVA patients ranged from 3.8 to 1,094 J/cm^2^. 

Of the 76 patients across the eight PUVA studies examined, 26 (34.2%) experienced complete remission of their skin lesions, and another 30 (39.5%) experienced partial remission (Table 2). Furthermore, several studies reported that patients achieved immunosuppression reduction post-therapy, demonstrating that managing cutaneous cGVHD is a crucial step in treating systemic disease [14-15, 31-35].

**Table 2:** Studies Describing PUVA Use for Cutaneous Chronic Graft-versus-host Disease (cGVHD) *One or more patients described this article did not fit the scope of our paper and were omitted from our analyses **This study included patients receiving UVB therapies (included in Table 4) † All six cutaneous cGVHD patients in Jampel 1991 were included in the list of the 34 cutaneous cGVHD patients in Vogelsang 1996 ‡ One patient had a short complete response that "rapidly relapsed with sclerodermoid GVHD" CR: complete remission; 8-MOP: 8-methoxypsoralen; OI: overall improvement; PR: partial remission; PUVA: psoralen and ultraviolet A; UVA: ultraviolet A; UVB: ultraviolet B

Author, Year	Type of cGVHD	Treatment Protocol	Average Cumulative Irradiation (Range) (in J/cm^2^)	Study Size	CR	PR	OI	Concomitant Immunosuppression	Reduction in Immunosuppression	Adverse Effects (number of patients)
Vogelsang et al. [13] 1996* & Jampel et al. [33] 1991†	Lichenoid, sclerodermatous	0.3 mg/kg of 8-MOP 1 hour before UVA irradiation (3 - 4x/wk, raised by 0.5 J/cm^2^ on alternating treatments as tolerated)	145.8 (5.5 - 1094)	34	13‡	9	22	31/33 on concomitant medications including prednisone, azathioprine, cyclosporine, thalidomide, methotrexate, or antithymocyte globulin	—	Severe phototoxicity (2/34), mild phototoxicity (4/40*), basal cell carcinoma after 7 years of PUVA (1/34), unspecified nausea due to 8-MOP
Ballester-Sánchez et al. [32] 2015**	Sclerodermatous, lichenoid, mixed, otherwise non-sclerodermatous	8-MOP dose unspecified, UVA irradiation at starting average 1.8 J/cm^2^ 2-3x/wk, raised by 0.5 J/cm^2^ every 2 - 3 sessions, up to average 4.4 J/cm^2^ 2-3x/wk	150 (unavailable)	10	3	7	10	Allowed, not specified	10/16 corticosteroid reduction**, 3/16 immunosuppressant reduction**	Erythema (6/16**), pruritus (1/16**)
Eppinger et al. [14] 1990*	Lichenoid, sclerodermatous	0.6 mg/kg of 8-MOP 2 hours before UVA irradiation, initial dose 0.3 - 1.0 J/cm^2^ at 4x/week, dose raised by 0.5 J/cm^2^ up to twice a week as tolerated to 3.5 - 7 J/cm^2^ max dosage. After resolution of skin symptoms, therapy 2x/wk and then 1x/week	95.8 (25.6 - 171)	7	3	4	7	Maintenance therapy 0.3 - 3 mg/kg prednisolone daily and additional azathioprine as needed	5/7 prednisolone reduction, 1/7 prednisolone cessation	Phrynoderma (9/11*) tolerable nausea, (4/11*)
Bonanomi et al. [15] 2001*	Lichen planus-like papulae and scleroderma, generalized follicular lichen planus-like eruptions and scleroderma, follicular lichen-planus-like	50 mL of 8-MOP (0.5% in 95% ethanol solution) mixed with 83 L of water. 20-minute 37° C bath before exposure to UVA (initially 0.3 - 0.5 J/m^2^ for 3x/wk, increase 0.3 - 0.5 J/m^2^ pending tolerance). Maintenance treatment 2x/wk and then 1x/wk for 6 - 12 months.	—	3	—	—	3	Mycophenolate mofetil, azathioprine, and cyclosporine for 2 patients, unknown for one patient	Tapering of systemic immunosuppression in two patients	Mild erythema (unspecified number)
Ghoreschi et al. [31] 2008	Sclerodermatous	30° C bath of 0.5 mg/L 8-MOP for 20 min before UVA (0.05 - 0.2 J/cm^2^ for 3 - 4x/wk, dose raised every third treatment by 0.1 - 0.5 J/cm^2 ^as tolerated). Five patients were given concomitant oral isotretinoin, 10 - 20 mg/day.	124.8 (7.5 - 505.4)	14	4	7	11	Methylprednisolone ≤ 20 mg daily	—	Skin ulcers on sclerodermatous lesions (11/14)
Leiter et al. [34] 2002	Lichenoid, sclerodermatous, pre-erythroderma	37° C bath of 0.5% 8-MOP for 20 min before UVA, dose increase dependent on skin type, administered 3 - 4x/wk in with breaks on days 3, 6, and 7 until improvement. Treatment then reduced to 2x/week and then 1x/week for the last 4 sessions.	26.7 (3.8 - 64.0)	6	3	3	6	6/6 prednisone, 5/6 mycophenolate mofetil, 1/6 cyclosporine	1/6 cessation in systemic therapy, 5/6 reduction in systemic therapy	Sunburn reactions (2/6)
Hoffner et al. [35] 2009	Erythematous sclerodermatous	Bath of 2.5 mg/L 8-MOP before UVA irradiation (0.3 J/cm^2^ for 3x/wk, increased 0.1 or 0.2 J/cm^2^ per session as tolerated).	91.2 (87.7 - 94.7)	2	—	—	2	2/2 deflazacort and mycophenolate mofetil	1/2 reduction in deflazacort	—

A retrospective study by Vogelsang et al. (with six patients previously reported in Jampel et al.) reported that 13 of 34 cutaneous cGVHD patients experienced complete remission of their skin lesions, and nine experienced partial reductions [13, 33]. Notably, patients with sclerodermatous cGVHD more often experienced partial (rather than complete) remission, and one sclerodermatous cGVHD patient with complete remission later experienced a relapse of their symptoms. Similarly, according to Ballester-Sánchez et al., out of 10 patients treated with PUVA, none of the sclerodermatous cGVHD patients experienced complete remission, whereas three patients with lichenoid cGVHD did [32]. Oral PUVA therapy may be more helpful in the treatment of lichenoid cGVHD rather than sclerodermatous cGVHD.

Compared to oral PUVA, bath PUVA may be more effective for sclerodermatous cGVHD. In Ghoreschi et al., a retrospective cohort study of 14 sclerodermatous cGVHD patients, four patients experienced complete remission, and seven had partial remission, offering some evidence that PUVA may be used to treat sclerodermatous cGVHD [31]. Two smaller studies have also reported treating sclerodermatous cGVHD using PUVA with varied results [34-35].

Both modalities demonstrated better outcomes for lichenoid cGVHD. Compared to oral PUVA, bath PUVA had a better rate of improvement for patients with sclerodermatous cGVHD, although 11 out of the 14 patients experienced ulcerations over their sclerodermatous lesions in the study by Ghoreschi et al. [31]. In general, the bath could be used to mitigate the systemic side effects of oral 8-MOP, including nausea. However, both oral and bath PUVA studies noted erythema and phototoxicity as side effects. Because of these toxicities and the declining numbers of facilities offering the treatment, PUVA has fallen out of favor among the phototherapies.

UVA1 irradiation

UVA1 refers to long-wave UVA (UVA1: 340 - 400 nm vs. UVA: 320 - 400 nm) and has been used in atopic dermatitis [1], cutaneous T-cell lymphoma [36], scleroderma [37], and other sclerosing skin diseases [38] for its ability to penetrate into the reticular layer of the dermis, which may make it more useful for sclerodermatous cGVHD. 

UVA1 irradiation has been shown to activate both cyclosporine A-sensitive and cyclosporine A-insensitive apoptosis pathways in T and B lymphocytes, triggering cell death by using constitutive intracellular apoptosis-initiating factors [39]. In contrast, PUVA and UVB (280 - 320 nm) cause delayed cell death reliant on the accumulation of proteins, such as p53 [38]. UVA1 also significantly decreases tumor necrosis factor-alpha levels after irradiation, whereas UVB results in significantly increased levels of the pro-inflammatory cytokine [40].

Treatment involves a body-length lamp that emits UVA1 light. In the context of cGVHD, patients receive therapy three to five times a week, starting at 10 - 50 J/cm^2^ of irradiation. In our review, the cumulative irradiation of patients receiving UVA1 ranged from 590 to 3,5000 J/cm^2^. Of the 52 patients treated with UVA1 across five studies, 44 (84.6%) experienced overall clinical improvement (Table 3) [16-17, 41-43].

**Table 3:** Studies Describing UVA1 Use for Cutaneous Chronic Graft-versus-host Disease *One or more patients described this article did not meet the scope of our paper and have been omitted from our analyses **Median cumulative irradiation cGVHD: chronic graft-versus-host disease; CR: complete remission; ECP: extracorporeal therapy; MMF: mycophenolate mofetil; OI: overall improvement; PR: partial remission; PUVA: psoralen and ultraviolet A; UVA1: ultraviolet A1

Author, Year	Type of cGVHD	Treatment Protocol	Average Cumulative Irradiation (Range) (in J/cm^2^)	Study Size	CR	PR	OI	Concomitant Immunosuppression	Reduction in Immunosuppression	Adverse Effects (number of patients)
Connolly et al. [16]*	Sclerodermatous	Typically 20 - 40 J/cm^2^, 40 - 80 J/cm^2^, or 80 - 120 J/cm^2^ of UVA1 for 2 - 3x/wk	3,165.4 (unavailable)	25	—	—	17	Allowed, not specified by individual patient	—	Erosion (1/25), erythema (1/25)
Wetzig et al. [17]*	Lichenoid, sclerodermatous	30 J/cm^2^ of UVA1 for 3 - 5x/wk, raised to a maximum of 60 J/cm^2^, as tolerated	1,330** (590 - 3,500)	10	6	3	10	10/10 cyclosporine, 6/10 methylprednisolone, 2/10 MMF, 2/10 ECP	3/10 cyclosporine tapered, 2/10 cyclosporine discontinued, 6/6 methylprednisolone discontinued, 1/2 MMF tapered, 1/2 MMF discontinued	Mild erythema (1/10)
Calzavara et al. [41]	Lichenoid, sclerodermatous	50 J/cm^2^ of UVA1 for 3x/wk	950 (600 - 1,650)	9	5	4	9	6/9 cyclosporine, 9/9 methylprednisolone, 3/9 MMF, 2/9 ECP, 1/9 azathioprine	9/9 reduction/discontinuation of immunosuppression	—
Ständer et al. [42]	Sclerodermatous	50 J/cm^2^ of UVA1 for 5x/wk, one patient received 1 treatment of 20 J/cm^2^, reduced to 3x/week after 2 months	—	6	—	—	6	Allowed, not specified	—	None
Ziemer et al. [43]	Lichenoid, sclerodermatous	20 J/cm^2^ 5x/wk to 50 - 60 J/cm^2^, 10 - 20 J/cm^2 ^5x/wk	990 (390 - 1,590)	2	0	2	2	Allowed, not specified	At least 1 discontinuation of immunosuppression	—

Calzavara et al. reported that of the five patients with sclerodermatous cGVHD, three experienced stable remissions and two achieved stable remission after another round of UVA1 treatment [41]. However, patients with lichenoid cGVHD experienced a relapse of their disease within a month of treatment cessation. All patients in the study reduced or discontinued their immunosuppression. However, the fact that lichenoid cGVHD patients required additional maintenance therapy suggests that, in contrast to PUVA, UVA1 therapy may be better suited for treating sclerodermatous cGVHD. A small case series of sclerodermatous cGVHD patients by Ständer et al. also reported improvement among patients, including increased joint mobility and healing of all lesions [42].

A study by Wetzig et al. showed that six out of seven lichenoid cGVHD patients undergoing UVA1 therapy experienced complete remission of their skin lesions, with one patient experiencing a relapse after 10 months [17]. The study also included three patients with sclerodermatous cGVHD who experienced either partial remission or “improvement of sclerotic skin lesions and joint mobility.” Nine of 10 cGVHD patients were able to reduce their immunosuppression regimen.

Connolly et al. reported a significant dose-dependent response to UVA1 therapy for sclerodermoid cGVHD patients; 12 patients (92.9%) on high-dose (80 - 120 J/cm^2^), four (50%) on medium-dose (40 - 80 J/cm^2^), and none on low-dose (20 - 40 J/cm^2^) responded to UVA1 therapy [16].

Throughout the studies, UVA1 demonstrated tolerability, with the most common side effects reported as erythema and erosion. However, the association between UVA1 and skin cancer risk has not been well-studied compared to that of UVB. Long-term adverse effects remain to be studied. Compared to PUVA, UVA1 therapy requires more frequent visits, usually three to five times per week, and the average cumulative irradiation dosage is multiple times higher. Due to the expense and shortage of facilities offering UVA1 therapy, it is less widely used.

UVB irradiation

UVB (280 - 320 nm) and narrowband (NB) UVB (311 - 313 nm) are used for the treatment of inflammatory skin diseases. A trial of 17 HSCT patients demonstrated that low-dose UVB irradiation effectively depleted CD1a+ Langerhans cells [44], which have been shown in mouse models to induce cutaneous graft-versus-host responses [45]. UVB has also been hypothesized to exert its anti-inflammatory effects through increased production of the immunosuppressive cytokine IL-10 in keratinocytes [46] and macrophages [47], which disrupts antigen presentation. Also, UVB exposure is associated with the production of 1,25-dihydroxyvitamin D_3_, which enhances the immunosuppressive activity of existing T regulatory (Treg) cells [48] and recruits more Treg cells by upregulating chemokine CCL22 [49].

**Figure 3:**
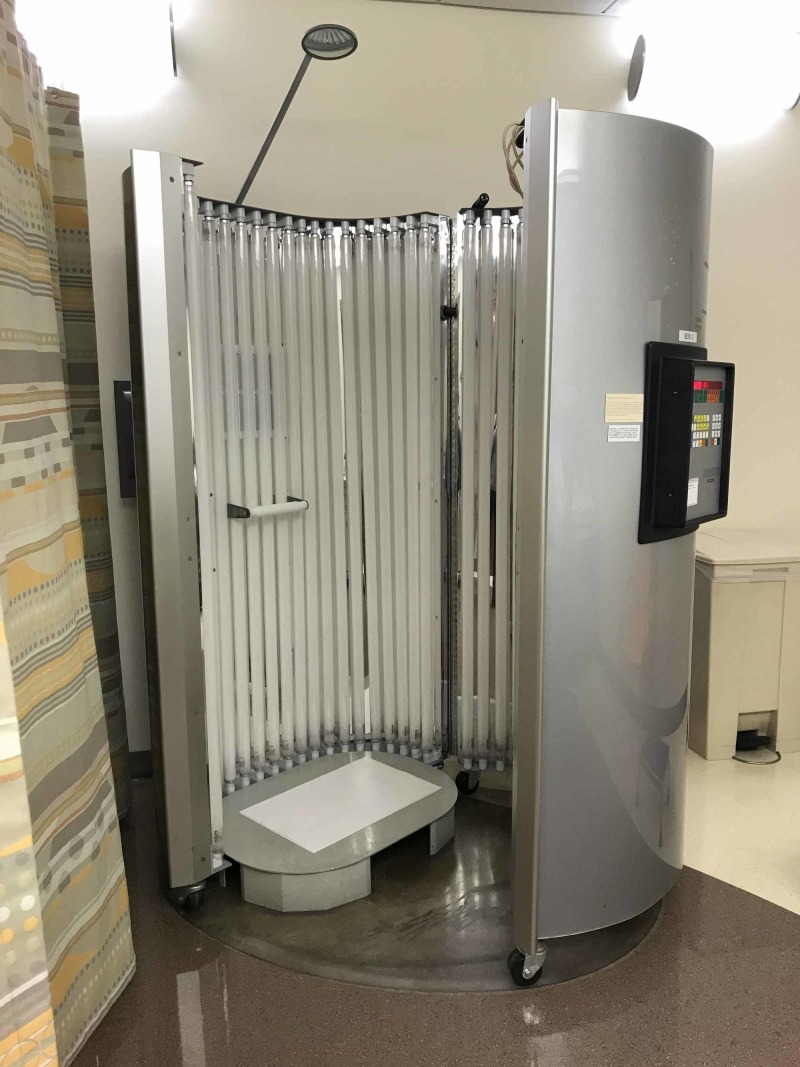
Ultralite full-body phototherapy system (Ultralite Enterprises, Inc., Dacula, GA)

UVB therapy is administered in a cabin lined by body-length panels of UVB-emitting lamps (Figure 3). The studies that were included in the review started at 0.035 - 0.25 J/cm^2^ of UVB two to three times a week and raised the dosage in increments of 0.02 mJ/cm^2^ to 0.05 J/cm^2^, or before the development of side effects, such as erythema [18-19, 32]. The cumulative dosing ranged from 1.02 - 70.38 J/cm^2^.

Of the 14 UVB patients in three publications analyzed, nine (64.3%) experienced complete remission of their cutaneous symptoms and four (28.6%) experienced partial remission (Table 4) [18-19, 32].

**Table 4:** Studies Describing UVB Use in Cutaneous Chronic Graft-versus-host Disease (cGVHD) *One or more patients described this article did not meet the scope of our paper and were omitted from our analyses **This study included patients receiving PUVA therapies (Described in Table 2) CR: complete remission; NB: narrowband; OI: overall improvement; PR: partial remission; PUVA: psoralen and ultraviolet A; UVB: ultraviolet B

Author, Year	Type of cGVHD	Treatment Protocol	Average Cumulative Irradiation (Range) (in J/cm^2^)	Study Size	CR	PR	OI	Concomitant Immunosuppression	Reduction in Immunosuppression	Adverse Effects
Brazzelli et al.* [18]	Lichenoid	0.035 - 0.18 J/cm^2^ of NB-UVB for 2 - 3x/wk, raised by increments of 0.05 J/cm^2^	29.3 (1.02 - 70.38)	5	4	—	4	Allowed, not specified	—	Erythema or pruritis (3/10*)
Ballester-Sánchez et al.** [32]	Lichenoid, mixed	0.25 J/cm^2^ of NB-UVB for 2 - 3x/wk, raised to an average max of 0.84 J/cm^2^	17 (not available)	6	4	2	6	Allowed, not specified	10/16 corticosteroid reduction**, 3/16 immunosuppressant reduction**	Erythema (6/16**), pruritus (1/16**)
Enk et al.* [19]	Lichenoid, sclerodermatous	Initial dose based on skin type, UVB for 2 - 3x/wk, raised by increments of 0.02 mJ/cm^2^ every two treatments	13 (5 - 24)	3	1	2	3	3/3 prednisone, 2/3 penicillamine, 1/3 thalidomide, 2/3 cyclosporine, 1/3 azathioprine, 1/3 chloroquine	—	Mild erythema (unspecified number)

Brazzelli et al. reported that out of five pediatric patients with lichenoid cGVHD treated with NB-UVB, four experienced complete remission of their skin lesions [18]. Similarly, Ballester-Sánchez et al. found that four of six patients treated with NB-UVB experienced complete remission, including patients with lichenoid and sclerodermatous presentations [32]. Enk et al. treated three patients with cutaneous cGVHD; one patient with lichenoid cGVHD experienced complete remission for up to 18 months, and two with sclerodermatous cGVHD experienced improvement of dryness and pruritus [19]. Unlike UVA1, UVB does not penetrate to the dermis, which is where sclerotic changes occur. This may explain the limited clinical response of patients with sclerodermatous cGVHD to UVB treatment.

Erythema was noted in all studies observed. No other phototoxicities were reported in the short-term, which points to UVB as a reasonable treatment for refractory lichenoid cGVHD. In all types of phototherapy, photoaging and long-term carcinogenicity must be considered. However, a literature review involving 11 studies with approximately 3,400 participants suggested that UVB phototherapy "remains a very safe treatment modality" [50].

## Conclusions

Topical and skin-directed therapies are integral components of treating cutaneous cGVHD. Many non-systemic treatments for cutaneous cGVHD are adapted from other T-cell-mediated skin disease therapies; however, many articles describing cutaneous cGVHD treatment are based on cases rather than randomized control trials. More research must be done to study skin-directed cGVHD treatments to ensure long-term safety and efficacy. Improvements in topical therapy would enable clinicians to better treat and manage the increasing number of patients diagnosed with cGVHD after undergoing allogeneic HCT.
